# Anomalous Liquid–Liquid Phase Separation Dynamics in Polymerization-Driven Complex Coacervation

**DOI:** 10.1021/acsmacrolett.6c00166

**Published:** 2026-06-11

**Authors:** Samiksha Shrivastava, Shensheng Chen

**Affiliations:** Department of Chemical and Biological Engineering, 58207 Hong Kong University of Science and Technology, Clear Water Bay, Kowloon, Hong Kong

## Abstract

Polymerization-driven liquid–liquid phase separation (LLPS) underpins critical biological processes and biomaterial innovations, yet its underlying kinetic pathway and domain growth law remain unclear. Here we report the first computational study of LLPS dynamics in polymerization-driven polyelectrolyte complex coacervation, where the degree of macromolecular charge asymmetry and polymer connectivity are dynamically changing during polymerization. We show that the polymerization-driven LLPS leads to two-stage kinetics in domain growth *L*(*t*): At an early stage, an exponential growth *L*(*t*) ∼ exp­(*t*) emerges due to polymerization-driven collapse of conjugated polyions; at later stage, the coupling between polymerization and coarsening lead to a single power-law in domain growth with near-linear scaling *L*(*t*) ∼ *t*. Both stages show drastically faster LLPS dynamics in domain growth compared to the classical prediction of *L*(*t*) ∼ *t* ^1/3^.

## Graphical abstract

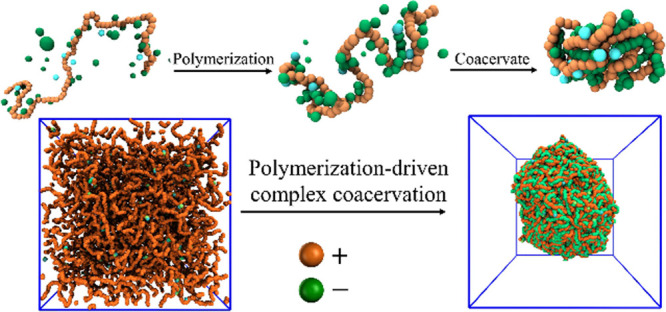

Polyelectrolyte complex coacervates are formed through electrostatics-driven liquid–liquid phase separation (LLPS) of oppositely charged polyelectrolyte solutions,^1,2^ resulting in a polymer-rich coacervate phase coexisting with a supernatant phase. In recent years, this LLPS process has attracted significant attention due to its biological relevance^3^ and biomaterial applications.^4,5^ Interestingly, in many scenarios, macromolecular LLPS requires environmental triggers such as pH,^6^ light,^7^ temperature,^8^ and reactants^9^ to activate one of the components to facilitate coacervation. These stimuli typically induce interspecies associations via ionization^10^ and polymerization^11^ in one of the components. In particular, polymerization-driven LLPS^12–15^ has emerged as an important strategy to control the self-assembly structures and functionalities of the resulting coacervates. For example, by polymerizing positively charged monomers into polycations at the presence of a polyanion solution, recent research^16^ demonstrates that such polymerization-induced LLPS can produce coacervate-based nanoreactors with controllable nanostructures. In addition, LLPS induced by physical-bonding polymerization is a promising method to create supramolecular materials.^17^

While recent efforts mostly focus on designing functional assembly using polymerization-driven LLPS, the corresponding nonequilibrium LLPS dynamics — including the kinetic pathway and domain growth law — remain poorly understood. LLPS dynamics determines the spatiotemporal evolution and thus have direct influence on the aging and the final structure of the coacervates. An in-depth study on the polymerization-driven LLPS dynamics is not only critical for advancing our understanding of the nonequilibrium LLPS physics, but also provides significant implications for the rational design of functional coacervate materials.

Current understanding of LLPS dynamics usually involves the concepts of droplet coarsening^18–20^ or Ostwald ripening,^21^ both lead to the universal Lifshitz–Slyozov-Wagner (LSW)^22^ scaling law, where the characteristic domain size *L*(*t*) grows with time as *L*(*t*) ∼ *t* ^1/3^.^22^ These theories do not incorporate the effects of electrostatic correlations and polymer connectivity of the charged polymers. Recent simulations^23^ show that, the macromolecular charge asymmetry between in polycation-polyanion droplets can significantly suppress their coarsening dynamics, leading to nonuniversal scaling law in domain growth. The polymer connectivity also has profound influence of LLPS dynamics: starting from a mixture of symmetric polycation and polyanion solutions, the connectivity nature of the polyions will lead to the formation of a polymer network at an early time of LLPS, with an intriguing *L*(*t*) ∼ *t* ^1/2^ scaling in domain growth.^24^ At a later time, the polymer network will in general break down, and the domain growth recovers to *L*(*t*) ∼ *t* ^1/3^ due to the coarsening of the discretized droplets.^25^

Introducing polymerization for one of the polyions (e.g., polymerizing polycations in the presence of polyanions, or vice versa, see the recent experiment^16^) will further complicate the problem, as it dynamically changes the degree of macromolecular charge asymmetry and polymer connectivity. It is not clear whether, or at what length scale, a universal scaling law in domain growth can be concluded in the polymerization-driven LLPS. So far, there are no theoretical or computational reports on the LLPS dynamics during polymerization-driven complex coacervation due to the complexity arising from the coupling of electrostatics, hydrodynamics, and polymerization.

In this work, we use Electrostatic Dissipative Particle Dynamics (EDPD) simulation^23,26,27^ to perform the first study of nonequilibrium LLPS dynamics in polymerization-driven complex coacervations. EDPD has the advantages of coupling the long-range hydrodynamic and electrostatic interactions, both of which are critical for the nonequilibrium LLPS dynamics. By implementing the atom-transfer radical polymerization (ATRP, a representative experimental polymerization method^28–30^ ) scheme into EDPD, we initiate the LLPS by polymerizing negatively charged monomers into polyanions in the presence of a polycation solution. Figure 1a–c schematically shows the ATRP process in our systems: The ATRP reaction begins by forming a covalent bond between an active initiator and a randomly selected negatively charged monomer within a cutoff. Consequentially, the connected monomer at the chain end will become the new active monomer, and the propagation continues (Figure 1a,b). The probability to form a bond, *P*_*p*_, will be a control parameter for polymerization-driven LLPS dynamics in this study. Figure 1c demonstrates that, under ATRP-polymerization of negatively charged monomers, a polycation will complex with the growing anionic chains and ultimately leads to the formation of a polycation–polyanion globule.

**Figure 1:**
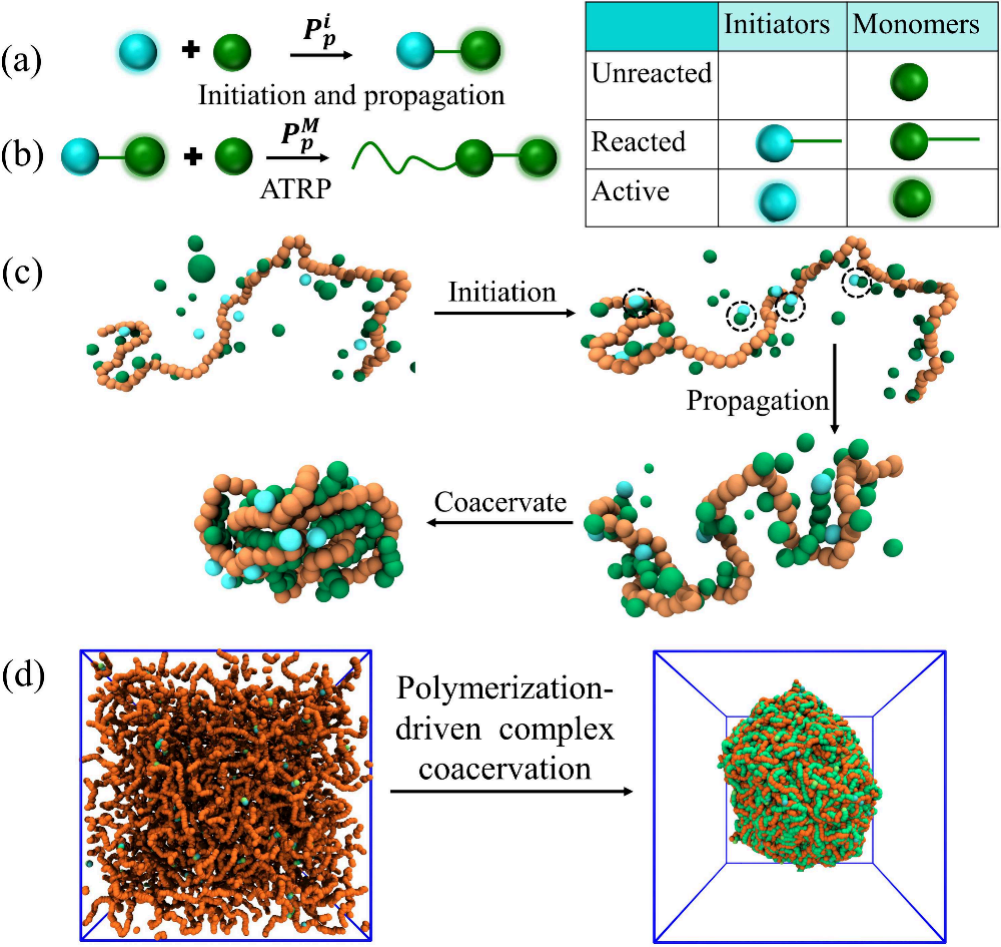
Schematic of Atom Transfer Radical Polymerization (ATRP) in the presence of polyelectrolytes. (a) Initiator activation. (b) Chain propagation with monomers. (c) Overview of the ATRP cycle in the presence of a polycation. The table details bead types. (d) The initial and final stage of polymerization-driven complex coacervation.

To investigate coarsening dynamics in polymerization-induced LLPS in semidilute solutions, we simulate a system with density ρ = 3*r*_*c*_^–3^ in a cubic box of dimensions 50 × 50 × 50 *r*_*c*_^3^, where *r*_*c*_ is the cutoff distance for DPD interactions and will serve as the reduced length unit of the simulation. All our systems contain *N*_+_ = 225 fully charged polycations with chain length *N*_*m* ^+^_ = 100, and *N*_*i* ^–^_ = 225 negatively charged initiators. In this charge symmetry system, the total negatively charged, reactive monomer (including initiators) concentration is fixed at *c*_*m* ^–^_ = 6.0%, corresponding to 22500 monomers. As such, the total charges are the same between polycations and anionic monomers. In addition, 750 small salt ions are added to the systems. Our initiator-to-monomer ratio of 1:100 (1.0 mol %) falls within the experimental range,^31–33^ where 0.1∼2 mol % are preferred for standard free radical polymerization processes. Simulating much lower initiator densities with enough overall monomers is beyond the capability of the current EDPD simulation method. Other simulation details are given in Supporting Information (SI). Figure 1d shows a typical example in this study where polymerization of polyanions in a sea of polycations result in the formation of a single large coacervate at the end.

We first investigate the domain growth dynamics in polymerization-driven LLPS in charge symmetric systems (*c*_*m* ^+^_ = *c*_*m* ^–^_ = 6.0%) under different *P*_*p*_ = 0.01, 0.03, 0.05, and 0.07, where *c*_*m* ^+^_ is the monomer concentration of polycations and *c*_*m* ^–^_ is the concentration of the reactive anionic monomers. In our simulation, *L*(*t*) is defined as the characteristic length scale using the first moment of the structure factor of polycations (see details in SI). In very early time up to *t*_0_, there is no coarsening since there are few polyanions being polymerized; the high charge asymmetry between the polycations and polyanions prevent phase separation.^23,34^ The coarsening starts to occur at time *t*_0_, which appears to occur at when the monomer conversion rate reach approximately 3% (Figure 3a) in all the cases. Interestingly, beyond *t*_0_, we clearly observe two distinct domain growth regions (Figure 2). The first region shows an exponential-like growth up to a time scale *t*_1_, i.e.,

$$L\left(t\right)\propto \exp\left(t\right),\mathrm{for}t_{0}<t<t_{1}$$ 1

This first-stage exponential growth is followed by a near-linear power-law growth as

$$L\left(t\right)\propto t,\mathrm{for}t>t_{1}$$ 2

The domain growth laws at both stages are very different from the traditional LSW scaling law of *t* ^1/3^.

**Figure 2:**
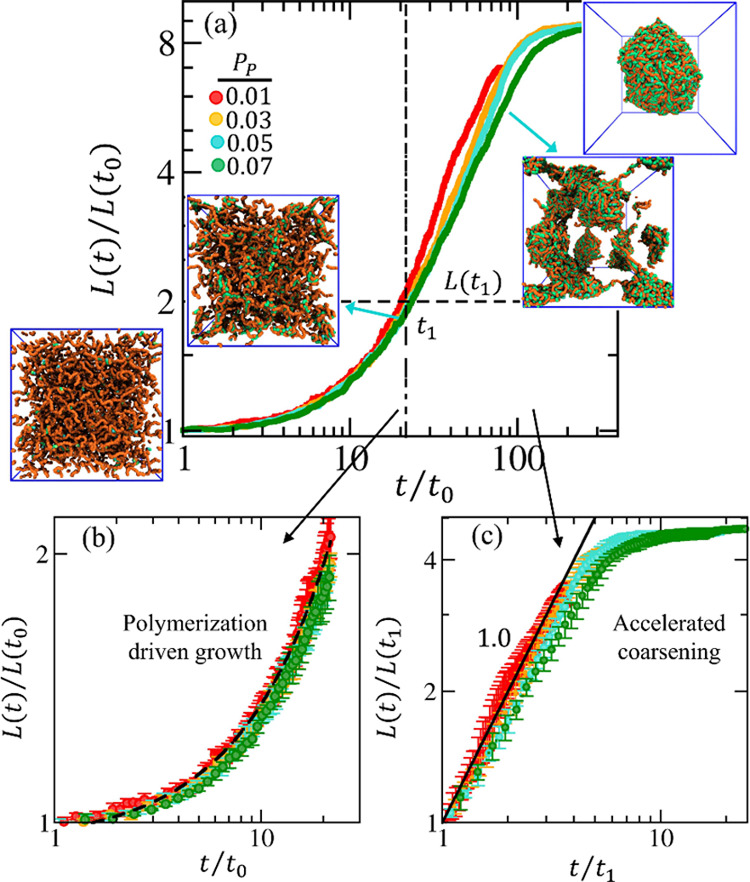
Symmetric case, *c*_*m*–_ = 6.0%, *c*_*m*+_ = 6.0%. (a) Characteristic length scale *L*(*t*)/*L*(*t*_0_) vs time *t*/*t*_0_ for propagation probabilities *P*_*p*_ = 0.01, 0.03, 0.05, 0.07. (b) Early stage *L*(*t*)/*L*(*t*_0_) vs *t*/*t*_0_ and (c) late-stage scaled growth, *L*(*t*)/*L*(*t*_1_) vs *t*/*t*_1_. Corresponding simulation snapshots are shown; solvent, salt, and unreacted monomers are omitted for clarity. The corresponding time evolution structure factor *S*(*q*, *t*) as a function of wave vector *q* are shown in SI, Figure S2.

In the early time (*t*_0_ < *t* < *t*_1_, with *t*_1_ being the time when monomer conversion rate reaches approximately 40%, Figure 3a), the polymerization of anions results in small multivalent oligomers that induce local strong binding with polycations, yet the high charge asymmetry between the oppositely charge polyions prevents macroscopic phase separations. Consequently, during this early stage, the domain growth is mostly driven by the collapse of polycations due to their local associations with multivalent anionic oligomers, as evidenced by the rapid decreasing in the polycation size shown in Figure 3c. Interestingly, in this stage, the binding between the polycations and anionic oligomers results in a local network-like structure (snapshots in Figure 2a). As the polymerization proceeds, the degree of charge asymmetry decreases, leading to a well-defined, continuous, and percolating network (Figure 2a). Subsequently, the domain growth *L*(*t*) accelerates, increasing from time *t*_0_ to *t*_1_ according to the power-law *L*(*t*) ∼ *t* ^*n*^ fit with increasing *n*. The measured exponents during this early time stage increases from (*n* = 0.16 at *t* = *t*_0_) to (*n* = 0.48) at *t* = *t*_1_. The upper limits of *n* in this early stage is close to networking-driven LLPS in complex coacervation, which yields *n* = 0.5.^24,25^ The dynamic increase of *n* in power-law leads to an exponential-like domain growth (Figure 2(b)). The exponential growth is more clearly justified as the straight lines in the log–linear plots, as shown in Figure S1 in Supporting Information. Since at this stage, the domain growth is mainly driven by polymerization-induced chain collapse and networking formation; we refer in stage as the polymerization-driven growth region.

**Figure 3:**
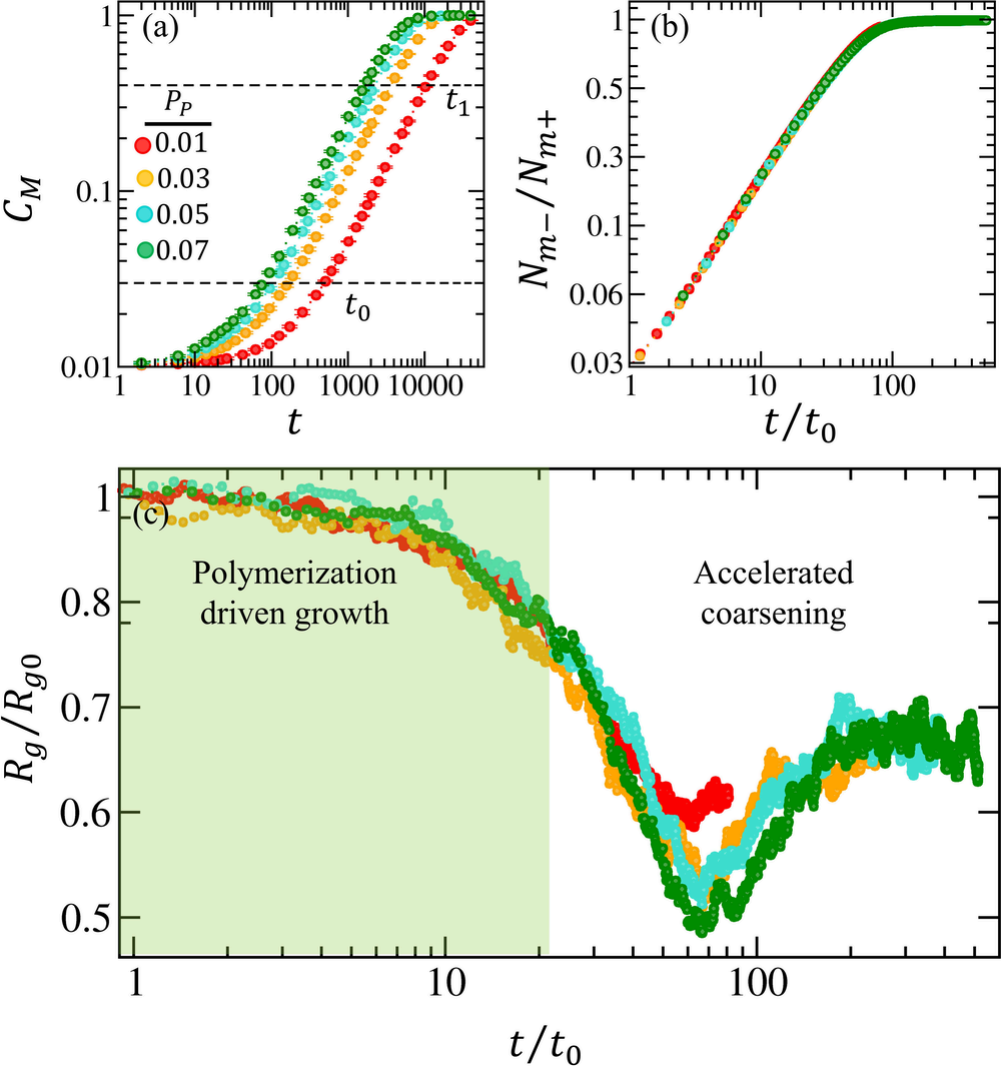
Symmetric case, *c*_*m*–_ = 6.0%, *c*_*m*+_ = 6.0%. (a) Monomer conversion *C*_*M*_ vs time *t*. (b) Average polyanion chain length *N*_*m*–_/*N*_*m*+_ vs time *t*/*t*_0_. (c) Radius of gyration *R*_*g*_/*R*_*g*0_ of polycation vs time *t*/*t*_0_.

Surprisingly, for later stage *t* > *t*_1_ when the monomer conversion increases more than *C*_*M*_ ≈ 40% (Figure 3 (a)), the domain growth clearly follow a single power-law scaling (Figure 2c) with an exponent close to 1.0. We note, *t*_1_ seems to occur at the same length scale for all *P*_*p*_, suggesting the transition occurs at a universal length scale, i.e., where the continuous network appears. Further verifications of this universal length scale requires testing broader range of *P*_*p*_, which is beyond the scope of this work. The rapid, single power law growth in this later stage indicates the dynamics is governed by interfacial tension-induced flow^20^ as the charge asymmetry decreases to a critical value (thus the interfacial tension increases). For *P*_*p*_ = 0.01∼0.07, the measured exponents in our simulations yields *n* = 1.07∼0.83. The observed near-linear power-law resembles the characteristics of the coarsening dynamics driven by viscous hydrodynamics.^35,36^ In simple binary fluid systems, the characteristic length scale of domain growth in the viscous-hydrodynamics region is predicted to follow *L*(*t*) ∝ *t*, which capture the kinetics of merging continuous domains driven by hydrodynamic pumping.^20,25^ Such a viscous hydrodynamic regime requires^20,37^

$${\left(D\eta \right)}^{1/2}\ll L\left(t\right)\ll \frac{\eta^{2}}{\rho \sigma }$$ 3

where *D* is the diffusion coefficient and σ is the surface tension. In eq 3, the lower bound denotes the crossover from diffusion regime to viscous regime. The upper bound requires the viscous force to dominate the inertial force, i.e., the Reynold’s number *R*_*e*_ = *ρuL*/η < 1. Since the velocity *u* scales with surface tension as *u* ∼ σ/η (which can be obtained by equating the viscous stress and Laplace pressure gradient in the viscous regime), the upper bound condition is justified, indicating a viscous-dominated regime. On the other hand, at much larger length scale of $L\left(t\right)>\frac{\eta^{2}}{\rho \sigma }$ , inertial forces dominate and the dynamics should follow *L*(*t*) ∼ *t* ^2/3^, characteristics of inertial-driven dynamics. So far, no MD simulations can reproduce the inertial-driven dynamics due to the large length scale required. It is widely know that the surface tension of coacervates is extremely low (on the order of 0.1% of the air–water surface tension) even at symmetric polycation–polyanion systems.^38–40^ The macromolecular asymmetry in this stage further decreases the surface tension,^23,27^ making the condition in the right-hand side of eq 3 naturally satisfied. The network formation drastically decreases free diffusion of the individual polymers.^24^ As such, if we take *D* as the polymer diffusivity in the network morphology, the left-hand side of eq 3 can also be easily satisfied. We note that eq 3 is only rigorously valid in simple binary fluids; Regardless, it provides a crude understanding of our complicated system.

Interestingly, the lower *P*_*p*_ value results in slightly faster exponents. We argue that the lower exponents in higher *P*_*p*_ are a result of faster network formation, as the polymerized oligomers will quickly connect to the polycations without allowing sufficient time for charge reorganizations. This is more clearly shown in the morphological evolution snapshots in Figure S3 in the Supporting Information. Since networking will lead to a transient *t* ^1/2^ scaling in domain growth before hydrodynamic pumping takes place^24,25^ the stronger network contributes to the slight slowdown of the linear scaling. As single power law growth is a signature of coarsening dynamics, we refer to this stage as the accelerated coarsening region. We note that the near-linear scaling spans to nearly one order of time magnitude when we increase the system size to 72^3^ *r*_*c*_^3^ (Figure S4 in Supporting Information). Due to the fact that the correlation length is independent of system size, we expect the near-linear scaling will persist with increased system size, until the length scale reaches the onset of the macroscopic, inertial hydrodynamic regime.

To better understand the crossover between the polymerization-driven and accelerated coarsening regions, we examine the evolution of monomer conversion and charge asymmetry between polyanions and polycations. Figure 3a shows that, at the early stage (*t*_0_ < *t* < *t*_1_), the domain growth is accompanied by a fast, power-law monomer conversion rate *C*_*M*_, which rapidly decreases the degree of charge asymmetry and constantly accelerate the domain growth. Indeed, Figure 3b demonstrates the ratio between polymerized polyanions and existed polycations, *N*_*m* ^–^_/*N*_*m* ^+^_, increases almost linearly until *t* = *t*_1_, which speeds up the polymerization-driven growth. The polymerization-driven nature in this early stage is more clearly shown in Figure 3c, where the radius of gyration of the polycations rapidly collapse for *t* > *t*_0_, due to their binding with the small anionic oligomers. For *t* > *t*_1_, as domains approach a critical characteristic size, both the *C*_*M*_ and *N*_*m* ^–^_/*N*_*m* ^+^_ slow down (Figure 3a,b), signaling that sufficient charge symmetry has been achieved to sustain domain growth. This slowdown marks the crossover from polymerization-driven into the coarsening-driven regime that ultimately gives way to the late-stage merging of continous domains driven by hydrodynamic pumping. At late time, this coarsening regime eventually transitions into a saturation plateau (coinciding with ⟨*N*_*m* ^–^_⟩/*N*_*m* ^+^_ ≈ 0.9), signaling the formation of stable coacervate droplets. In this overall charge symmetry setup, most systems (except the system of *P*_*p*_ = 0.01 where not all the monomers are polymerized at the final simulation time) results in the same final domain size with the formation of a single droplet (Figure 2).

We expect that the networking pathway and near-linear scaling is unique to the electrostatic-driven polyelectrolyte complex coacervation, due to the tendency to bridge positive and negative polymers during polymerizations. To this end, we compare our results with the polymerization-driven kinetics of neutral polymer systems under poor solvent conditions, with other simulation setup similar to our charged systems (see simulation details in SI). As shown in Figure S5 in Supporting Information, in the neutral systems, the polymerized chains quickly collapse into small condense globules, and the phase separation mainly proceeds as droplet coarsening. Consequently, the domain growth in the neutral systems follows *L*(*t*) ∼ *t* ^1/3^, as expected from the classical LSW theory for droplet coarsening. While in our polyelectrolyte systems, the low surface tension and the tendency of macromolecular charge neutrality leads to the formation of a continuous network, resulting in *L*(*t*) ∼ *t* ^1^ due to hydrodynamic pumping. Although an apple-to-apple comparison between segregated phase separation (the neutral system) and associative phase separation (our charged system) is not straightforward, this comparison highlights the unique pathway and kinetics in electrostatics-driven polyelectrolyte phase separation during polymerizations. Furthermore, if we initiate the system with well-mixed polycations and polyanions under the same overall monomer concentration as in our polymerization system, the domain growth under this nonpolymerization system show the classical *t* ^1/3^ scaling, as shown in Figure S6 in Supporting Information. This result once again highlights the role of polymerization in the network formation and the corresponding hydrodynamic pumping driven kinetics.

We next examine whether the LLPS dynamics is influenced by the charge asymmetry arising from the initial concentrations of the reactive anionic monomers. To this end, we studied a system at a fixed cationic monomer concentration (*c*_*m* ^+^_ = 6.0%, same as previous case) in polycations, with a higher concentration of anionic reactive monomers (*c*_*m* ^–^_ ≈ 11.9%). Additional positively charged monomers are added to the system to maintain global charge neutrality. Figure 4a shows that, at each *P*_*p*_, *L*(*t*) shows similar qualitative behavior to the case of the symmetric systems: A polymerization-driven exponential growth occurs at early time *t* > *t*_0_, followed by a single power-law coarsening growth will exponent close to 1.0; Systems with higher *P*_*p*_ will result in slightly smaller exponents.

**Figure 4:**
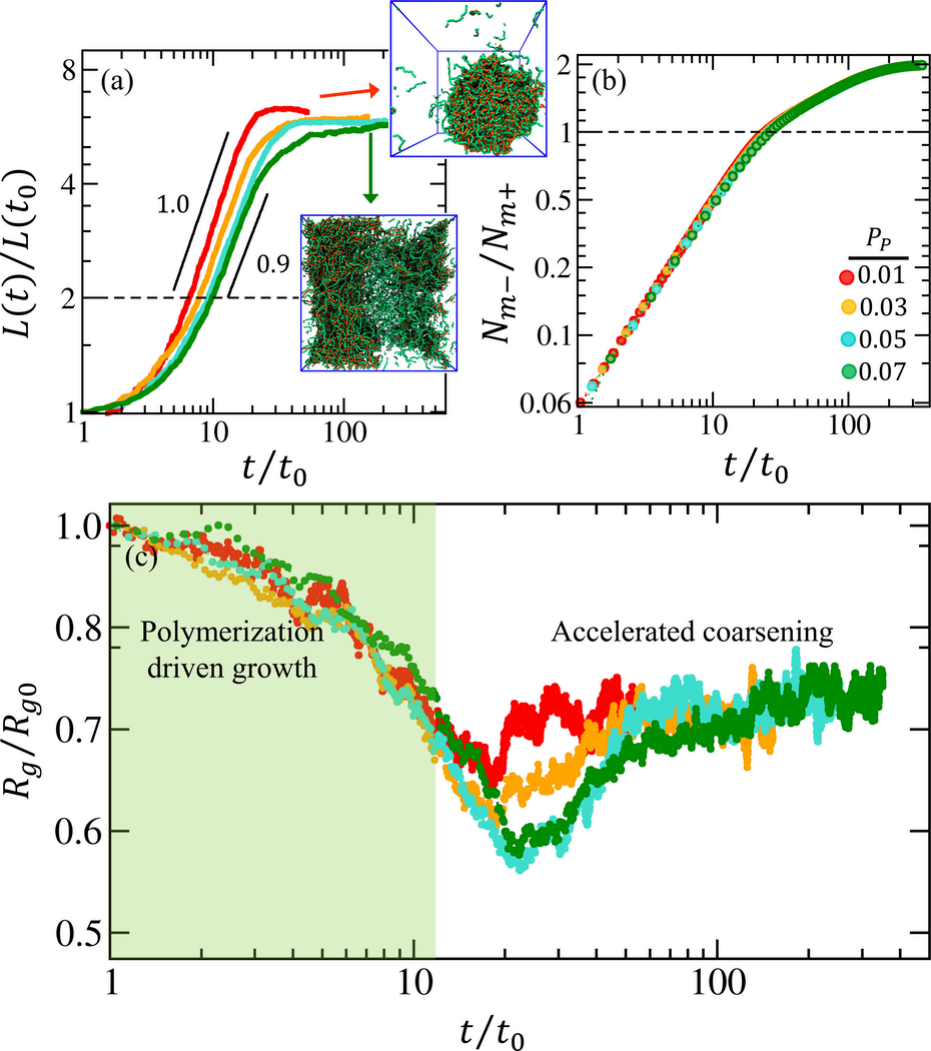
Asymmteric case, *c*_*m* ^–^_ = 12%, *c*_*m* ^+^_ = 6.0%. (a) *L*(*t*)/*L*(*t*_0_) vs scaled time *t*/*t*_0_ for propagation probabilities *P*_*p*_ = 0.01, 0.03, 0.05, and 0.07. (b) Average polyanion chain length *N*_*m* ^–^_/*N*_*m* ^+^_ vs time *t*/*t*_0_. (c) Radius of gyration *R*_*g*_/*R*_*g*0_ of polycation vs time *t*/*t*_0_. Corresponding simulation snapshots are shown; solvent, salt, and unreacted monomers are omitted for clarity.

Compared to the (overall) charged symmetric case in Figure 2, some interesting quantitative differences emerge. First, the polymerization-driven growth starts to occur at slightly higher total monomer conversion (approximately double of the symmetric case, see Figure S7) due to the stronger electrostatic screening effects caused by higher concentrations of small ions. In this asymmetric case, however, the domain growth is faster overall, with a shorter period for the polymerization-driven growth (Figure 4 a). This accelerated growth is a consequence of faster monomer conversion, driven by a more available reactive monomer pool, as shown in Figure 4b.

Perhaps more interestingly, in the charge asymmetry (*c*_*m* ^–^_ > *c*_*m* ^+^_) systems, higher *P*_*p*_ will lead to smaller domain size at the end. In the case of small *P*_*p*_ = 0.01, the final morphology is a single cluster; however, in high *P*_*p*_, the polymerization-driven LLPS leads to an arrested network, as shown in the snapshots of Figure 4. This is due to the fact that, at later stage, there are more polyanions (via polymerization) than polycations, i.e., charge inversion occurs, as evidenced in Figure 4b. Consequently, the net macromolecular charges in the polymer clusters prevent them from complete coarsening.^23^ As shown in Figure S7, higher *P*_*p*_ leads to faster charge inversion, therefore leads to stronger suppression on phase separation and smaller final domain size. A series of representative morphological evolution snapshots are shown in Figure S8 for the cases of *P*_*p*_ = 0.01 and *P*_*p*_ = 0.07. On the other hand, if the overall polymerized anion monomers are less than the pre-existed polycations, the macromolecular charged asymmetry will likely leads to arrested dynamics at later stage, with the formation of multiple net-charged droplets as the final state.^23^

In summary, we demonstrate that polymerization-driven polyelectrolyte complex coacervation will result in anomalous dynamics in domain growth that is distinctively different from the classical *t* ^1/3^ scaling law. At early time, polymerization of small anions into short oligomers induces local interchain correlations that collapses the existed polycations. The continuous decrease of polymer charge asymmetry couples to the collapse of the polycations leads to an exponential increase in domain growth. As the polymerization proceed to decrease the charge asymmetry to a critical value at *t* = *t*_1_, the domain growth starts to follow a single power law with an exponent close to 1.0, where faster *P*_*p*_ results with slightly smaller exponents. When the total anionic and cationic monomers are equal (*c*_*m* ^–^_ = *c*_*m* ^+^_), for all the polymerization rates we tested, all the systems evolve into a single droplets at the end. When there are more polymerizable anionic monomer than polycation monomer (*c*_*m* ^–^_ > *c*_*m* ^+^_), polymerization leads to charge inversion at a later stage, where faster *P*_*p*_ leads to smaller final domain size.

This work represents our initial efforts to employ EDPD for simulating nonequilibrium LLPS dynamics driven by polymerization of charged monomers and highlights the significant role of polymerization in regulating LLPS pathway and kinetics. Although we focus on idealized flexible polyelectrolytes and the resulting complex coacervates, we expect the general physics elucidated in this work will be relevant to broader biocondensates driven by electrostatic correlation of various charged biomacromolecules that involve polymerization. More studies are warranted to explore other effects of LLPS dynamics such as salt concentrations, chain structures, and charge distributions.

We note that the overall polymer/monomer concentration of our work is apparently over the overlap polymer concentration. Therefore, our systems are in the semidilute polymer solution regime.^41^ With further increased polymer concentrations, we expected the network formation and the hydrodynamic-pumping growth to persist, as it is easier to form a network at high concentrations. On the other hand, at much lower concentrations, (i.e., for dilute polymer solutions), there are not enough chains to form a network during polymerization, and the corresponding LLPS are expected to proceed as droplet coarsening. Finally, we note that due to the high computational cost, our linear growth regime can only persist over approximately 1 order of magnitude. Thus, we cannot completely rule out the possibility that this near linear scaling is a result of artifact regime around an inflection point sitting between the initial and final stages. However, given the different scaling laws we obtained from neutral and nonpolymerization systems, as well as the physical rational of hydrodynamic pumping, we believe our near linear scaling is robust with system size.

## Supplementary Material



## Data Availability

Data will be available upon request.

## Notes

Other simulation details; Electrostatic dissipative particle dynamics simulation; Atom transfer radical polymerization; Morphology characterization function, including supporting figures: Figure S1 for log–linear plot of domain growth; Figure S2 for the structure factor *S*(*q*, *t*) as a function of the wave vector *q*; Figure S3 for evolution snapshots for a symmetric case; Figure S4 for domain growth for 72^3^ *r*_*c*_^3^ and 50^3^ *r*_*c*_^3^; Figure S5 for domain growth law in polymerization-driven phase separation of neutral polymer system; Figure S6 for domain growth *L*(*t*) in the absence and in the presence of polymerization; Figure S7 for monomer conversion vs time for asymmetric case; Figure S8 Evolution snapshots for asymmetric case (PDF)
