# Supplementary material for: Anomalous Liquid–Liquid Phase Separation Dynamics in Polymerization-Driven Complex Coacervation
Source: ACS Macro Lett. 2026 Jun 11;15(7):962–7. doi: 10.1021/acsmacrolett.6c00166 (PMC13394411; doi:10.1021/acsmacrolett.6c00166)
Supplement: Supplementary file 1 [file mz6c00166_si_001.pdf]

Supplementry information: Anomalous Liquid-Liquid Phase Separation Dynamics in Polymerization-Driven Complex Coacervation

Samiksha Shrivastava and Shensheng Chen*

Department of Chemical and Biological Engineering

Hong Kong University of Science and Technology

E-mail: shensheng@ust.hk

Other simulation details:

Electrostatic Dissipative Particle Dynamics Simulation (EDPD)

In our EDPD simulation, monomers of polycations and monoanions, salt, and the solvent (water) are explicitly modeled by coarse-grained beads having the same mass m and size r_c. Following the classical setup in DPD,[1] the net force f_i on the ith bead by the rest of jth bead within cut-off distance r_c is given by:$\vec{f}_i = \sum_{i \neq j} \left[\vec{F}^C_{ij} + \vec{F}^D_{ij} + \vec{F}^R_{ij} + \vec{F}^E_{ij} \right]$ we set the non-bonded interaction as $F^c_{ij} = a_{ij}(1 - r_{ij}/r_c)\hat{r}_{ij}$, which is a linear soft repulsive force between i^{th} and j^{th} beads. In this equation, a_{ij} is the strength of the interaction, $r_{ij} = |r_i - r_j|$ is the inter-bead distance, and $\hat{r}_{ij} = (r_i - r_j)/r_{ij}$ represents the direction of the force. In DPD, the cut-off distance r_c is considered as an intrinsic length scale, and k_BT as the energy scale of the system. a_{ij} is the maximum repulsion between the beads, thereby

influencing the structural and dynamical characteristics of the system.[1,2] $\vec{F}_{ij}^D$ and $\vec{F}_{ij}^R$ are the dissipative and random forces, respectively. The mathematical expression of these forces are given by: $\vec{F_{ij}}^D = -\gamma\omega^D(r_{ij})(\hat{r}_{ij} \cdot \vec{v}_{ij})\hat{r}_{ij}$, $\vec{F_{ij}}^R = \sigma\omega^R(r_{ij})\xi_{ij}\hat{r}_{ij}$. Here, γ and σ denote the the strength of dissipative and random forces (friction coefficient and noise coefficient), respectively and $\vec{v}_{ij} = \vec{v}_i - \vec{v}_j$ is the relative velocity between i^{th} and j^{th} beads. Random variable ξ_{ij} is taken as Gaussian distributed with zero mean and unit variance:[1–3] $\langle \xi_{ij}(t) \rangle = 0$, and $\langle \xi_{ij}(t)\xi_{kl}(t') \rangle \, (\delta_{ik}\delta_{jl} + \delta_{il}\delta_{jk})\delta(t - t')$. Here, the local momentum conservation can be ensured by the symmetry property $\xi_{ij} = \xi_{ji}$. The direction of these forces is also along line of bead centers $\hat{r}_{ij}$. To satisfy the fluctuation-dissipation theorem (FDT), the strength of dissipative and random forces is given by: $\gamma = \frac{\sigma^2}{2k_BT}$, and the weight functions are paired with the relation:[1,2,4] $\omega^D(r_{ij}) = \left[\omega^R(r_{ij})\right]^s = 2\sigma k_B T$. The simulation time step is set to $\Delta t = 0.02\tau$ where $\tau = (mr_c^2/k_BT)^{1/2}$ is defined as the characteristic time scale. The total bead number density in the simulation box is set to $\rho = 3$.

The electrostatic force between two charged DPD beads i and j is given by $\vec{F}_{ij}^E = \frac{Cq_iq_j}{\epsilon r^3}\left[1 - e^{-2r/\lambda}\left(1 + \frac{2r}{\lambda} + \frac{2r^2}{\lambda^2}\right)\right]\vec{r}_{ij}$. Where q_i and q_j are the charges, ϵ is the dielectric constant, λ is the Slater screening length, and $C = 1/(4\pi\epsilon_0)$ is the Coulomb constant. To focus on electrostatic interactions, we set repulsion a_{ij} between all species as $a_{ij} = 25k_BT/r_c$, representing good solvent conditions for the polymers. m, k_BT, r_c are respectively the mass, energy and length units in DPD simulations, which are all set to unity. We model polyelectrolytes as linear chains of charged beads (either $+e$ or $-e$), accompanied by oppositely charged counterions. Salt and reactive anionic monomers are modeled as monovalent charged beads. In this work, we use the following charge distribution for DPD particles:[5–8]

$$\rho(r) = \frac{q}{\pi\lambda^3}e^{-2r/\lambda}, \tag{1}$$

where $\lambda = 0.25$ is the decay length of the charge distribution, and the Coulombic cutoff distance is set to $r_{\text{coul}} = 4.5$. Integration of Eq. (1) over the entire space yields the total charge

q. This parameterization balances the accuracy of long-range electrostatics and computation efficiency. Standard DPD pair-wise thermostat[1] is used in this work, which conserves local momentum and captures correct hydrodynamics.

Polymer chains are modeled by the widely used bead-spring model, where the beads (monomers) within each chain are connected by the harmonic bond potential as $E_b = \frac{1}{2}k_b(r - r_0)^2$. Here, $k_b = 128$ is the elastic bond strength and $r_0 = 0.5$ is the equilibrium bond length between consecutive beads.[9,10] To avoid bond crossing,[11,12] we add a small angle potential as: $E_a = \frac{1}{2}k_a(\cos\theta - \cos\theta_0)^2$, where $k_a = 5$ is the potential strength and $\theta_0 = 180°$ is the equilibrium angle.

Atom Transfer Radical Polymerization (ATRP)

In the ATRP process, the initiator and monomer are modeled as DPD beads. In this process, the reaction begins by randomly selecting the initiator beads, followed by the random selection of monomer beads within an interaction radius of $r_i = 0.7r_c$ for the formation of potential covalent bonds. To reproduce the linear first-order kinetics characteristic of living radical polymerization,[11,13,14] we set the cutoff radius to $r_i = 0.7r_c$, with $r_c = 1$ in reduced DPD units. Smaller values of $r_i < 0.7r_c$ led to delayed polymerization relative to experimental observation, while larger values $r_i > 0.7r_c$ resulted in noticeable deviations from first-order kinetic behaviour. The probability of polymerization $0 < P_r^a < 1$, where the superscript a denotes the type of reaction $a = i$ for the initiation and $a = M$ for the propagation of the monomer, determines whether a bond will form. For each reaction step, a uniformly distributed random number $n_r \in (0, 1)$ is drawn and compared with P_r^a. If $n_r < P_r^a$, the reaction is accepted, leading to an irreversible covalent bond modeled by a harmonic potential. This stochastic approach ensures controlled chain growth while maintaining the dynamic nature of the polymerization process. The details of the bead types involved in the primary reaction steps are summarized via a schematic shown in Fig.1 in the main text. The integration time step in the simulation is set to $\Delta t = 0.02\tau$. In this work, the

reaction between any two successive reaction steps is selected to be $\tau_r = 0.2\tau$, i.e., the reactions are performed every ten simulation time steps.[10,14] Note that we consider only the translation motion of the particle and ignore the rotational motion for simplicity. In the context of the ATRP process, we have excluded both degenrative chains transfer and active radical termination reaction lead to the conversion of active radicals into dromant soecies. In general, the polymerization rate is several orders of magnitude smaller than the diffusion rate of monomers beads in the system.[11,13,15] Thus, polymerization kinetics is a slow process, which implies that ATRP is a kinetically controlled reaction. In our polymerization scheme, monomer addition occurs every τ_r/P_p time interval.[11,13] The time scale for the addition of one monomer in a chain can be estimated as $\tau_r/P_p \approx 3\tau \sim 20\tau$. Given the monomer diffusion time scale in simulation is around $\tau_0 \simeq 1\tau$, and the chain diffusion time scale increased with chain length as $\tau_0 N^2$ for semidiute polymer solutions, our polymerization rate is much slower the diffusive time scale, as expected real ATRP processes. Our ATRP simulation setup is consistent with previous work.[11,13,14]

Morphology Characterization Function

We calculate domain evolution via the structure factor $S(q,t)$ at time t:

$$S(q,t) = \frac{1}{N_b} \sum_{i}^{N} \sum_{j}^{N} \langle \exp\left[i\boldsymbol{q} \cdot (\boldsymbol{r}_i(t) - \boldsymbol{r}_j(t))\right] \rangle, \tag{2}$$

where $\boldsymbol{q}$ is the wave vector with magnitude q, N_b is the total number of polycation beads, and $\boldsymbol{r}_i$ and $\boldsymbol{r}_j$ are the positions of bead i and j, respectively. The characteristic wave number $\langle q \rangle$ at time t is calculated by the first moment of $S(q,t)$:

$$\langle q(t) \rangle = \frac{\int q S(q,t) dq}{\int S(q,t) dq}, \tag{3}$$

The characteristic domain size at time t is then given as $L(t) = 2\pi/\langle q(t) \rangle$.

Supporting figures

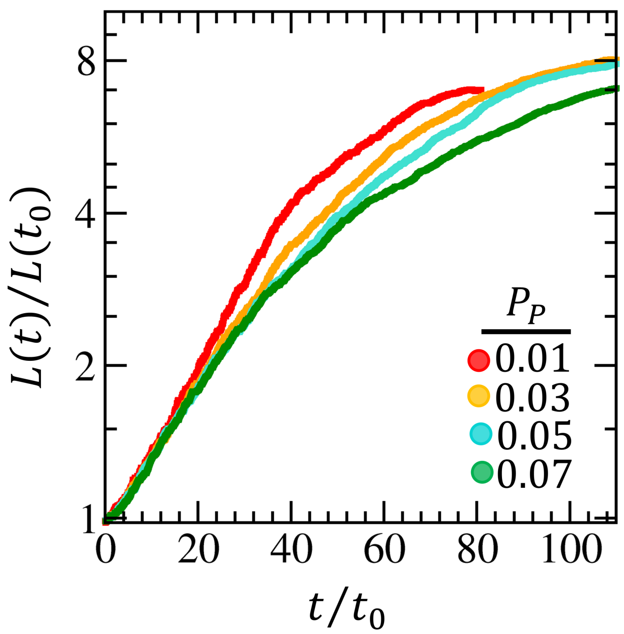

Fig. S1: Log-linear plot of domain growth, where t_0 is the time when 3% of monomers are polymerized

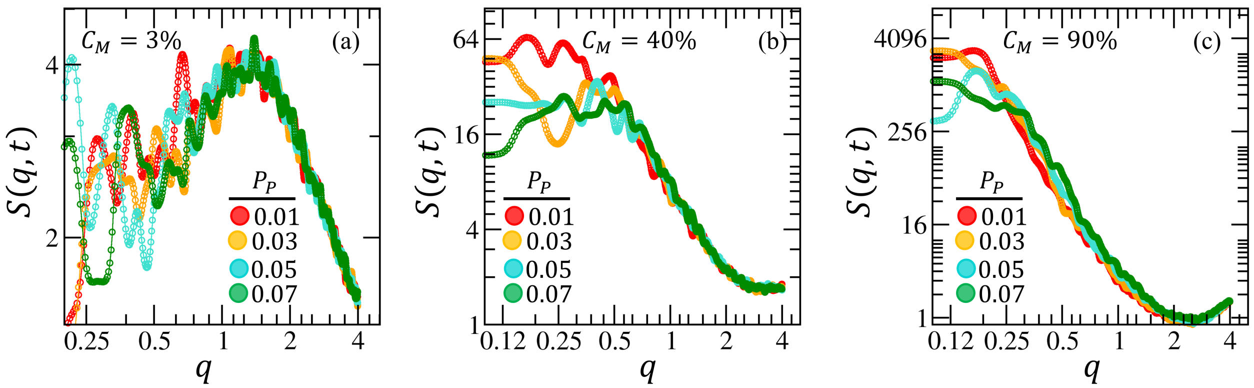

Fig. S2: Structure factor $S(q,t)$ as a function of the wave vector q for propagation rates $P_p = 0.01, 0.03, 0.05, 0.07$. Results are shown for monomer concentrations (a) $C_M = 3\%$, (b) 40%, and (c) 90%.

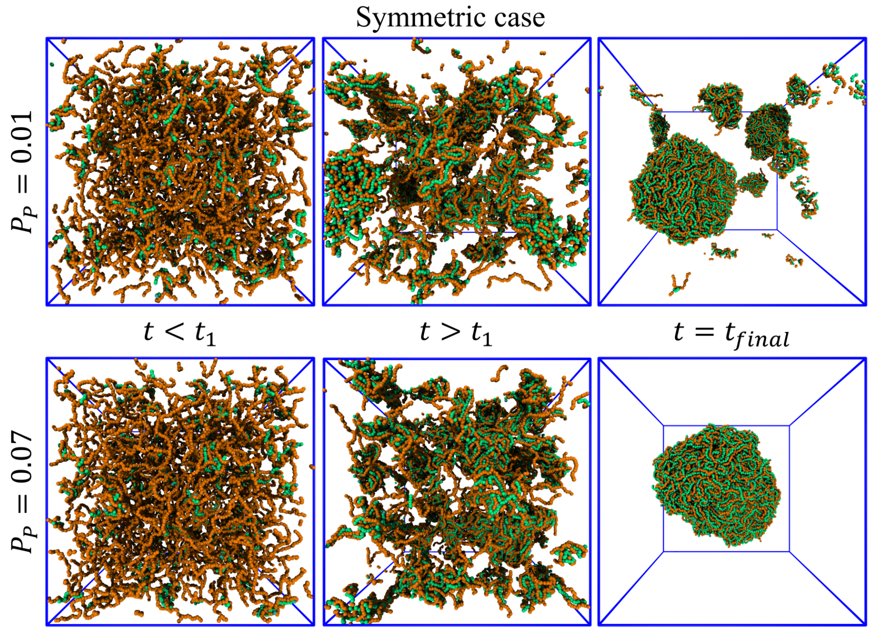

Fig. S3: Evolution snapshots during polyanion polymerization in the presence of polycations at different times for charge symmetric systems: (a) early stage ($t < t_1$), (b) intermediate stage ($t > t_1$), and (c) final configuration ($t = t_{\text{final}}$) for propagation rates $P_p = 0.01$ and $P_p = 0.07$.

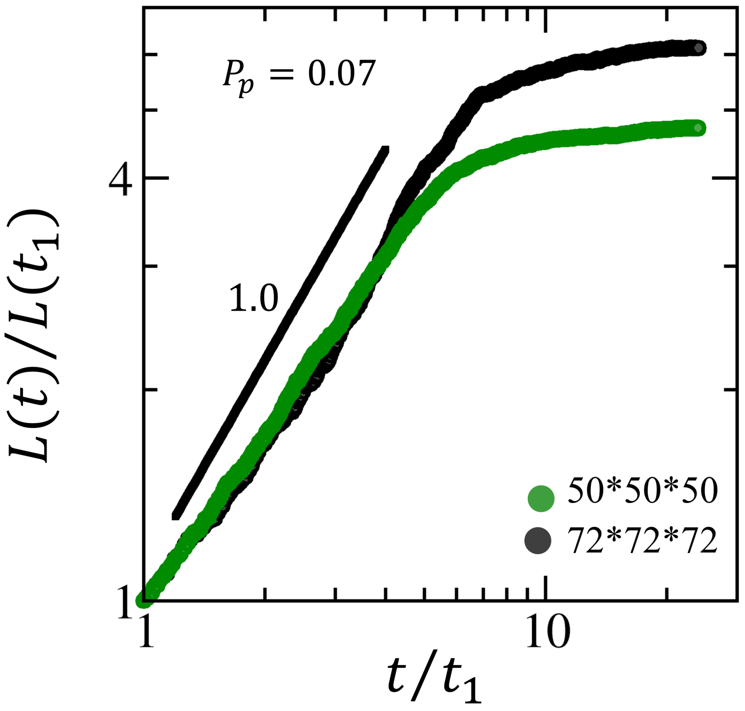

Fig. S4: Domain growth in much larger simulation box $72^3 r_c^3$ and in $50^3 r_c^3$

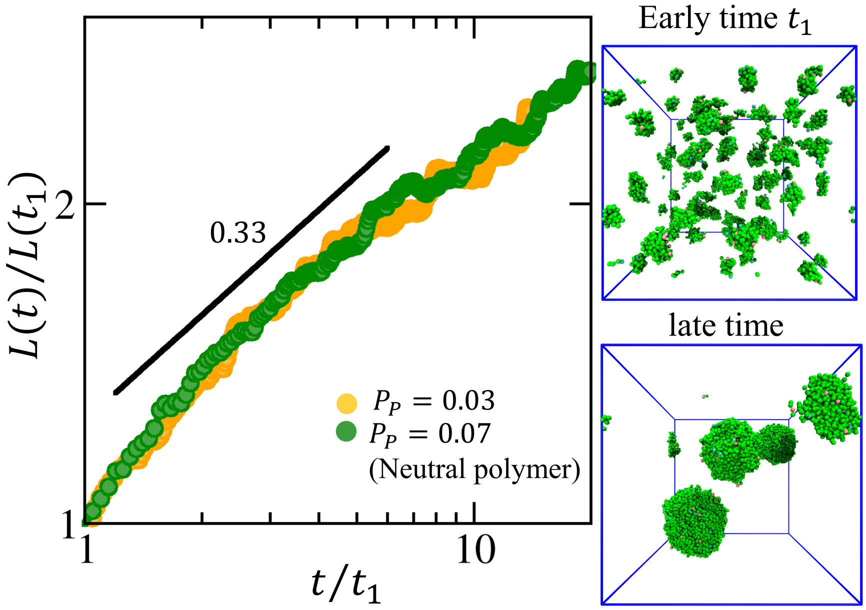

Fig. S5: Simulation snapshots and domain growth law in polymerization-driven phase separation of neutral polymer system with polymer-solvent repulsion $a_{ij} = 32$ for propagation probability $P_p = 0.03$, and 0.07

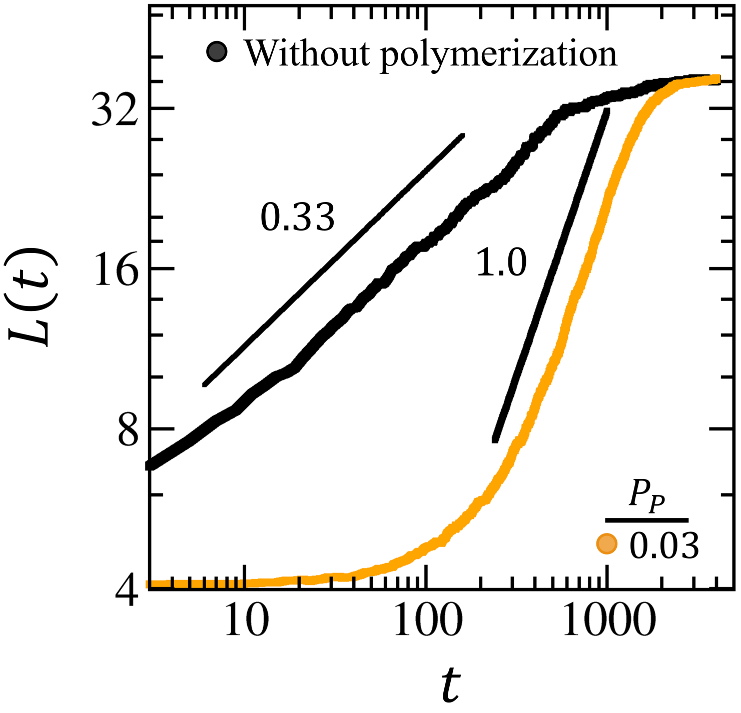

Fig. S6: Comparison of characteristic domain growth $L(t)$ in the absence of polymerization (both polycations and polyanions are pre-mixed) and in the presence of polymerization with propagation probability $P_p = 0.03$.

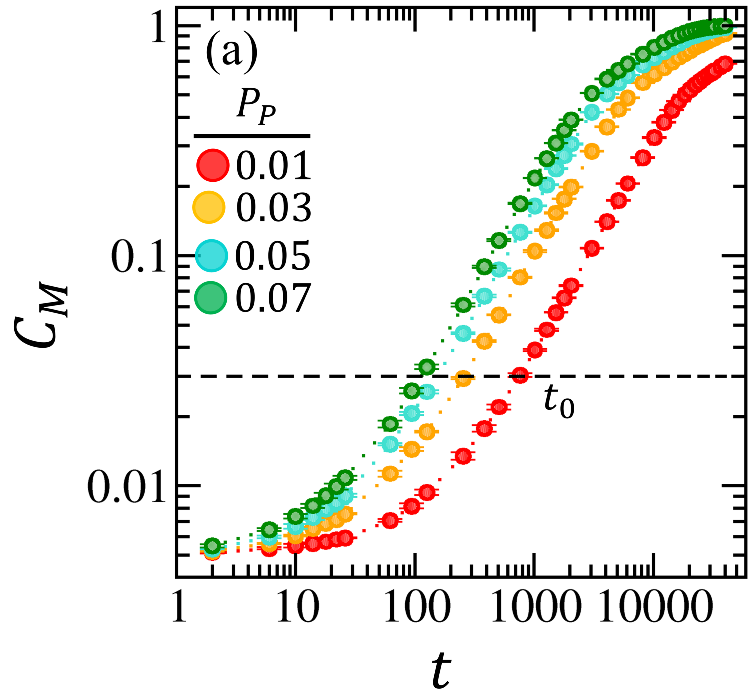

Fig. S7: Monomer conversion C_M vs. time t in asymmetric case with $c_{m-} = 11.9\%$, $c_{m+} = 6.0\%$.

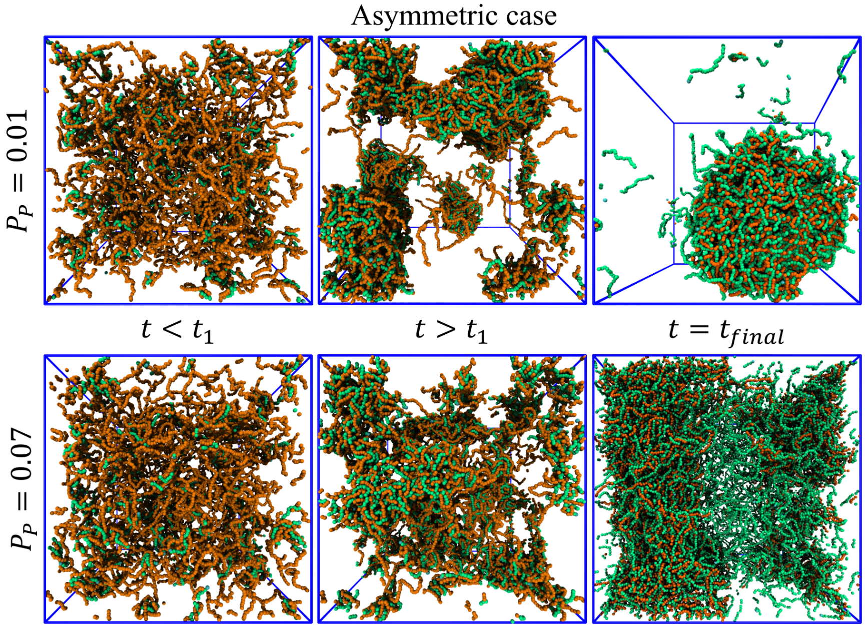

Fig. S8: Evolution snapshots during polyanion polymerization in the presence of polycations at different times for charge asymmetric systems: (a) early stage ($t < t_1$), (b) intermediate stage ($t > t_1$), and (c) final configuration ($t = t_{\text{final}}$) for propagation rates $P_p = 0.01$ and $P_p = 0.07$.

References

(1) Groot, R. D.; Warren, P. B. Dissipative particle dynamics: Bridging the gap between atomistic and mesoscopic simulation. *The Journal of chemical physics* **1997**, *107*, 4423–4435.

(2) Espanol, P.; Warren, P. Statistical mechanics of dissipative particle dynamics. *EPL (Europhysics Letters)* **1995**, *30*, 191–196.

(3) Espanol, P.; Warren, P. B. Perspective: Dissipative particle dynamics. *The Journal of chemical physics* **2017**, *146*.

(4) Nikunen, P.; Karttunen, M.; Vattulainen, I. How would you integrate the equations of motion in dissipative particle dynamics simulations? *Computer physics communications* **2003**, *153*, 407–423.

(5) González-Melchor, M.; Mayoral, E.; Velázquez, M. E.; Alejandre, J. Electrostatic interactions in dissipative particle dynamics using the Ewald sums. *The Journal of chemical physics* **2006**, *125*.

(6) Saint-Martin, H.; Hernández-Cobos, J.; Bernal-Uruchurtu, M. I.; Ortega-Blake, I.; Berendsen, H. J. A mobile charge densities in harmonic oscillators (MCDHO) molecular model for numerical simulations: the water–water interaction. *The Journal of Chemical Physics* **2000**, *113*, 10899–10912.

(7) Chen, S.; Zhang, P.; Wang, Z.-G. Complexation between oppositely charged polyelectrolytes in dilute solution: Effects of charge asymmetry. *Macromolecules* **2022**, *55*, 3898–3909.

(8) Chen, S.; Wang, Z.-G. Charge asymmetry suppresses coarsening dynamics in polyelectrolyte complex coacervation. *Physical Review Letters* **2023**, *131*, 218201.

(9) Singh, A.; Kuksenok, O.; Johnson, J. A.; Balazs, A. C. Photo-regeneration of severed gel with iniferter-mediated photo-growth. *Soft Matter* **2017**, *13*, 1978–1987.

(10) Shrivastava, S.; Saha, S.; Singh, A.; others Dissipative particle dynamics simulation study on ATRP-brush modification of variably shaped surfaces and biopolymer adsorption. *Physical Chemistry Chemical Physics* **2022**, *24*, 17986–18003.

(11) Yong, X.; Kuksenok, O.; Matyjaszewski, K.; Balazs, A. C. Harnessing interfacially-active nanorods to regenerate severed polymer gels. *Nano letters* **2013**, *13*, 6269–6274.

(12) Chen, S.; Olson, E.; Jiang, S.; Yong, X. Nanoparticle assembly modulated by polymer chain conformation in composite materials. *Nanoscale* **2020**, *12*, 14560–14572.

(13) Yong, X.; Kuksenok, O.; Balazs, A. C. Modeling free radical polymerization using dissipative particle dynamics. *Polymer* **2015**, *72*, 217–225.

(14) Ifra; Singh, A.; Saha, S. High Adsorption of α-Glucosidase on Polymer Brush-Modified Anisotropic Particles Acquired by Electrospraying—A Combined Experimental and Simulation Study. *ACS Applied Bio Materials* **2021**, *4*, 7431–7444.

(15) Ifra; Singh, A.; Saha, S. Shape shifting of cup shaped particles on growing poly (2-hydroxy ethyl methacrylate) brushes by “Grafting From” approach and dissipative particle dynamics simulation. *ChemistrySelect* **2020**, *5*, 4685–4694.
